# Effect of Exogenous Melatonin on Corn Seed Germination and Seedling Salt Damage Mitigation Under NaCl Stress

**DOI:** 10.3390/plants14071139

**Published:** 2025-04-06

**Authors:** Yuyu Zhang, Yuchuang Li, He Liu, Haili Xie, Jiani Liu, Jinzhu Hua, Mingchun Xiong, Huaifei Song, Chengjian Yong

**Affiliations:** 1 College of Agriculture and Life Sciences, Kunming University, Kunming 650214, China; miechelle@126.com (Y.Z.); lychuan72@163.com (Y.L.); hel151872@163.com (H.L.); 15912382293@139.com (H.X.); jinzhuh@163.com (J.H.); 2 Yunnan Characteristic Resource Plants Intelligent Agriculture Engineering Center, Kunming 650214, China; 3 Yunnan Jiayuanshi Biotechnology Co., Ltd., Kunming 650214, China; xiongmingchun@biogaeder.cn; 4 Agricultural and Rural Work Service Centre, Haiping Street, Shuicheng District, Liupanshui 553000, China; scqsgb3_2@sina.com

**Keywords:** melatonin, maize, salt damage, mitigation, photosynthesis, antioxidant system

## Abstract

Maize is very sensitive to salt stress during seed germination and seedling growth periods, which can seriously affect the development of the maize industry. In this study, we applied exogenous melatonin (MT) to treat maize seeds and seedlings to investigate the alleviation mechanism of salt damage in maize. Phenotypic analyses showed that 100 µmol/L MT alleviated the effects of salt stress on maize seed germination, and germination index and vigor index were increased compared with salt treatment. MT also alleviated the effects of salt stress on biomass and photosynthesis of maize seedlings, and at a concentration of 100 µmol/L, root and shoot lengths were increased, Gs and Tr were significantly elevated, and LWUEint and LWUEins were decreased. MT also scavenged ROS accumulation, reduced MDA, H_2_O_2,_ and O_2_^−^ production, and increased antioxidant enzyme activities and osmoregulatory substances in maize seedlings, but too high a concentration exacerbated oxidative and osmotic stresses. In addition, MT reduced Na^+^ content and increased K^+^ content in leaves and roots of maize seedlings. The principal components analysis explained 99.1% of the total variance in the first two axes (PC1 and PC2), and the differences between the treatment groups along the PC1 and PC2 axes were obvious. Correlation analysis elucidated the correlation between the indicators. Random forest analysis showed that different treatments had significant effects on germination percentage (GP), free proline (FP), CAT, and leaf intrinsic water use efficiency (LWUEint). Partial least squares analysis showed that photosynthetic parameters and pigment content played an important role in the salt tolerance of maize seedlings. In conclusion, the application of exogenous MT can effectively alleviate the negative effects of salt stress on the growth of maize seeds and seedlings, especially at a concentration of 100 µmol/L, which is the most effective.

## 1. Introduction

Maize (*Zea mays* L.) is an important food crop [1]. China is the second largest producer of maize in the world, and its production accounts for 23.5% of the total global grain production. Thus, it ensures global food security. [2]. Although maize is one of the extremely high-yielding crops, it is more susceptible to abiotic stresses such as climate change and soil salinization than other cereal crops [3,4]. Currently, maize is severely endangered by salinity, which restricts growth and productivity [5]. This hazard is global, especially in arid and semi-arid regions where salt stress is more severe due to natural (topography and matrices, low rainfall, high evapotranspiration) and anthropogenic (inappropriate fertilizer application and irrigation) factors [2,6,7]. Salt stress triggers many types of metabolic disorders in plants, including osmotic stress, oxidative stress, and ionic imbalance, which can further induce physiological drought, ionic toxicity, and nutrient deficiencies, ultimately leading to crop failure [5,8,9,10,11]. For example, salt stress induces ionic imbalance by inhibiting effective K^+^ uptake, leading to unfavorable mineral uptake and resulting in mineral deficiency [12,13,14,15]. Salt stress prevents plants from efficiently absorbing and utilizing water, leading to impaired water uptake and reduced leaf water and osmotic potentials, resulting in physiological drought [13,16,17]. Salt stress also induces the production and accumulation of reactive oxygen species (ROS), which damages cell membrane lipids, proteins, nucleic acids, and photosynthetic systems [5,6,9,18,19]. Worse still, salt stress destroys chloroplast structure and the activities of photosynthesis-related enzymes, ultimately leading to yield stagnation [2,20]. Therefore, it is extremely urgent to take measures to mitigate the effects of salt stress on maize production.

In recent years, various substances have been widely used to improve the salt tolerance and yield of crops [5,6,9,21,22]. Melatonin (MT), an indole small molecule widely found in plants and animals, has been found to have a variety of biological functions, especially playing an important role in plant growth and development and response to abiotic stress [23,24,25,26,27,28,29,30,31,32]. Thus, foliar spraying and root irrigation showed significant alleviation of salt stress resistance in rice (*Oryza sativa* L.) [33]. It has also been shown that its application to soybean, wheat, maize, and rice crops confirmed the ability of MT to alleviate salt stress-induced growth retardation and promote seed germination and root development [34,35,36,37]. Likewise, MT effectively scavenges ROS, reactive nitrogen species (RNS), free radicals, and harmful oxidative molecules and acts as a signaling molecule to enhance the antioxidant enzymes superoxide dismutase (SOD), catalase (CAT), and peroxidase (POD) [38,39,40,41,42]. MT also promotes the production of salt-tolerant proteins and defense-associated molecules, thereby protecting the potato (*Solanum tuberosum* L.) from the deleterious effects of abiotic stresses [43]. Under salt stress, 70 µM MT treatment improved photosynthetic efficiency, increased chlorophyll content, and reduced ROS production in barley (Streptococcus aureus) plants, thereby alleviating plant oxidative damage. [44]. MT significantly increased the leaf area, biomass, and photosynthetic efficiency of mustard-type oilseed rape under 150 µM NaCl stress [45]. Altogether, exogenously applied MT may be a way to alleviate stress injury in a wide range of plants [46].

Currently, most of the studies on exogenous MT to cope with growth regulation under abiotic stress have been focused on rice [33], potato [43], barley [44], and oilseed rape [45], whereas there are few reports around the application of different concentrations of exogenous MT on maize seed germination and seedling salt damage under salt stress. Therefore, in this study, we used a dominant maize variety (YR668) as the material, simulated salt stress with NaCl (100 mM), and applied exogenously fading MT by dipping and watering to investigate the effects of different concentrations of exogenous MT on maize seed germination and seedling growth under salt stress, aiming to alleviate soybean salt damage and provide valuable references for the selection of excellent salt-tolerant maize varieties, the improvement of saline soil, and how to maintain corn yield to provide valuable references.

## 2. Results

### 2.1. Effect of MT on Maize Seed Germination Under NaCl Stress

**Figure 1:**
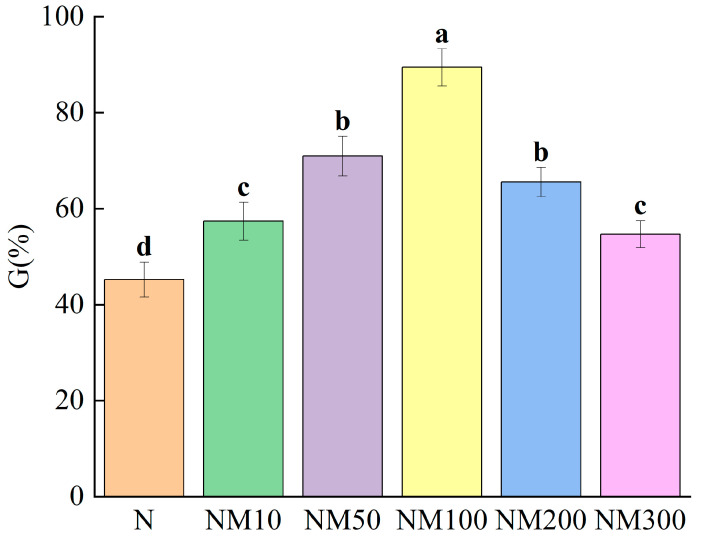
Effect of MT on Maize Seed germination under NaCl Stress. Data are shown as means ± SD. Different letters indicate the significant difference among different treatments at the significant level of *p* < 0.05, as determined by one-way ANOVA. G%: the germination percentage; N: NaCl; NM10: 150 mmol NaCl + 10 µM MT; NM50: 150 mmol NaCl + 50 µM MT; NM100: 150 mmol NaCl + 100 µM MT; NM200: 150 mmol NaCl + 200 µM MT; NM300: 150 mmol NaCl + 300 µM MT.

Salt stress inhibited the germination of maize seeds, while different concentrations of MT affected the germination percentage of maize seeds (Figure 1). Among them, NM100 had the greatest effect on germination percentage, which was 1.98 times higher than that of the N treatment (*p* < 0.05). As the concentration of MT increased, the germination percentage gradually decreased, and under NM 300, the germination percentage was 61.13% of that of NM100. The results indicated that there was a dose effect of the MT, with low concentrations promoting the germination of maize seeds under salt stress and high concentrations inhibiting it.

### 2.2. Effect of Exogenous MT on the Seedling Growth Indicators of Maize Under NaCl Stress

Salt stress significantly inhibited the growth of maize seedlings, while MT promoted the growth of maize seedlings (Table 1). Among them, NM100 had the greatest effect on seedling growth indexes (*p* < 0.05), which were 5.40, 5.42, 2.83, 2.65, 2.67, 2.23, and 1.51 times higher than N, respectively, in that order. However, the increase in MT concentration rather inhibited the growth of maize seedlings, and the root length, shoot length, root fresh weight, shoot fresh weight, root dry weight, shoot dry weight, and seedling leaf area of NM300 were 38.36%, 52.20%, 48.53%, 50.00%, 62.50%, 43.75%, and 72.55% lower than that of NM100, respectively. Thus, MT had a dose effect on the growth of maize seedlings under salt stress, with low concentrations of MT alleviating the inhibitory effect, while high concentrations exacerbated the inhibition.

### 2.3. Effects of Exogenous MT on Photosynthetic Parameters of Maize Seedlings Under NaCl Stress

**Figure 2:**
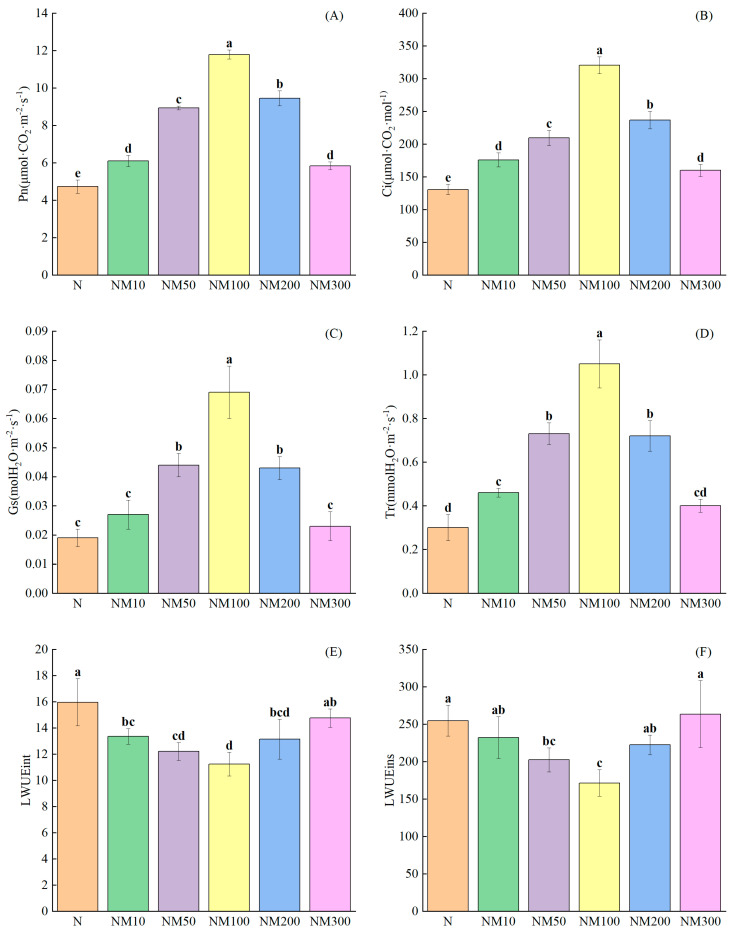
Effects of Exogenous melatonin (MT) on Photosynthetic Parameters of Maize Seedlings under NaCl Stress. The error lines in the figure represent the standard deviation (SD) based on three replicates (*n* = 3). Different letters indicate the significant difference among different treatments at the significant level of *p* < 0.05, as determined by one-way ANOVA. (**A**) Pn: photosynthetic rate; (**B**) Ci: intercellular CO_2_ concentration; (**C**) Gs: stomatal conductance; (**D**) Tr: transpiration rate; (**E**) LWUEint: leaf intrinsic water use efficiency; (**F**) LWUEins: leaf instantaneous water use efficiency. N: NaCl; NM10: 150 mmol NaCl + 10 µM MT; NM50: 150 mmol NaCl + 50 µM MT; NM100: 150 mmol NaCl + 100 µM MT; NM200: 150 mmol NaCl + 200 µM MT; NM300: 150 mmol NaCl + 300 µM MT.

Photosynthetic parameters can reflect the physiological status and changes in maize seedlings under salt stress [47]. Salt stress significantly inhibited photosynthesis, decreasing Pn (52.32%), Ci (50.34%), Gs (63.46%), and Tr (64.29%) (*p* < 0.05) (Figure 2). Application of exogenous 50 µM MT was effective in alleviating the damage of photosynthesis by salt stress and increased Pn (88.79%) and Ci (60.2%). NM100 had the greatest effect on Gs and Tr, which were increased by 263.16% and 250%, respectively (Figure 2C,D). However, higher concentrations of MT resulted in a decrease in these indices, but they were still higher than N, except that Tr decreased by 42.38% in the NM300 treatment (Figure 1). Moreover, LWUEint and LWUEins were significantly increased by 34.54% and 32.16% (*p* < 0.05), respectively, with no treatment (Figure 2E,F), but the application of exogenous 100 µM MT decreased LWUEint and LWUEins by 29.56% and 32.73%, respectively.

### 2.4. Effects of Exogenous MT on Chlorophyll Accumulation of Maize Seedlings Under NaCl Stress

**Figure 3:**
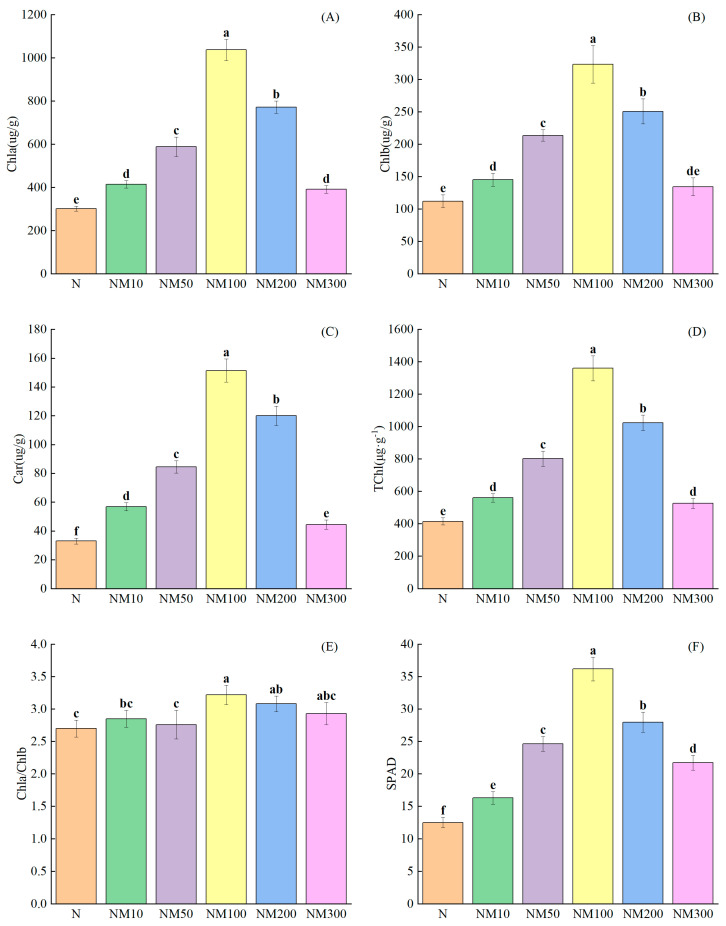
Effects of Exogenous melatonin (MT) on Chlorophyll Accumulation of Maize Seedlings under NaCl Stress. The error lines in the figure represent the standard deviation (SD) based on three replicates (*n* = 3). Different letters indicate the significant difference among different treatments at the significant level of *p* < 0.05, as determined by one-way ANOVA. (**A**) Chla: chlorophyll a; (**B**) Chlb: chlorophyll b; (**C**) Car: carotenoids; (**D**) Tchl: total chlorophyll content; (**E**) Chla/Chlb: ratio of chlorophyll a to chlorophyll b; (**F**) SPAD: the relative chlorophyll content. N: NaCl; NM10: 150 mmol NaCl + 10 µM MT; NM50: 150 mmol NaCl + 50 µM MT; NM100: 150 mmol NaCl + 100 µM MT; NM200: 150 mmol NaCl + 200 µM MT; NM300: 150 mmol NaCl + 300 µM MT.

Salt stress significantly inhibited the accumulation of chlorophyll (*p* < 0.05), such as reduced levels of Chla (67.75%), Chlb (63.46%), carotenoids (67.91%), Tchl (66.69%), Chla/Chlb (11.48%), and SPAD (62.07%) (Figure 3). Exogenous 100 µM MT was effective in mitigating the deleterious effects of salt stress by significantly increasing chlorophyll accumulation in maize seedlings by 243.35%, 188.18%, 359.54%, 228.41%, 19.26%, and 189.21%, respectively (*p* < 0.05). However, the ratio of chlorophyll a to chlorophyll b slightly increased by 7.85% under NM300 treatment (Figure 3E).

### 2.5. Effect of Exogenous MT on Cell Membrane Damage of Maize Seedlings Under NaCl Stress

**Figure 4:**
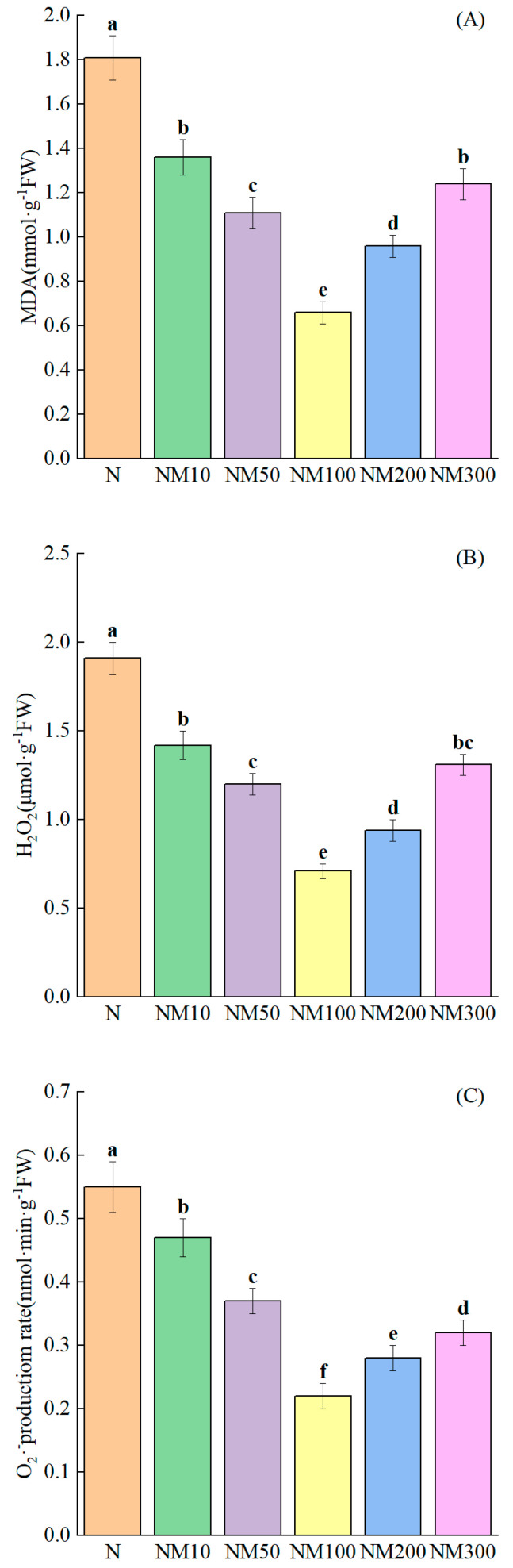
Effect of Exogenous melatonin (MT) on cell membrane damage of Maize Seedlings under NaCl stress. The error lines in the figure represent the standard deviation (SD) based on three replicates (*n* = 3). Different letters indicate the significant difference among different treatments at the significant level of *p* < 0.05, as determined by one-way ANOVA. (**A**) MDA: malondialdehyde; (**B**) H_2_O_2_: hydrogen peroxide; (**C**) O_2_^−^: the rate of superoxide anion production; N: NaCl; NM10: 150 mmol NaCl + 10 µM MT; NM50: 150 mmol NaCl + 50 µM MT; NM100: 150 mmol NaCl + 100 µM MT; NM200: 150 mmol NaCl + 200 µM MT; NM300: 150 mmol NaCl + 300 µM MT.

Salt stress caused severe damage to the cell membranes of maize seedlings, increasing MDA, H_2_O_2,_ and O_2_^−^ contents by 123.46%, 127.38%, and 120.83%, respectively (*p* < 0.05) (Figure 4). Compared with salt stress, exogenous MT attenuated the oxidative damage by 24.86–63.54%, 25.65–62.83%, and 14.55–60%, respectively, especially at an MT of 100 µM.

### 2.6. Effects of Exogenous MT on Antioxidant Enzyme Activities of Maize Seedlings Under NaCl Stress

**Figure 5:**
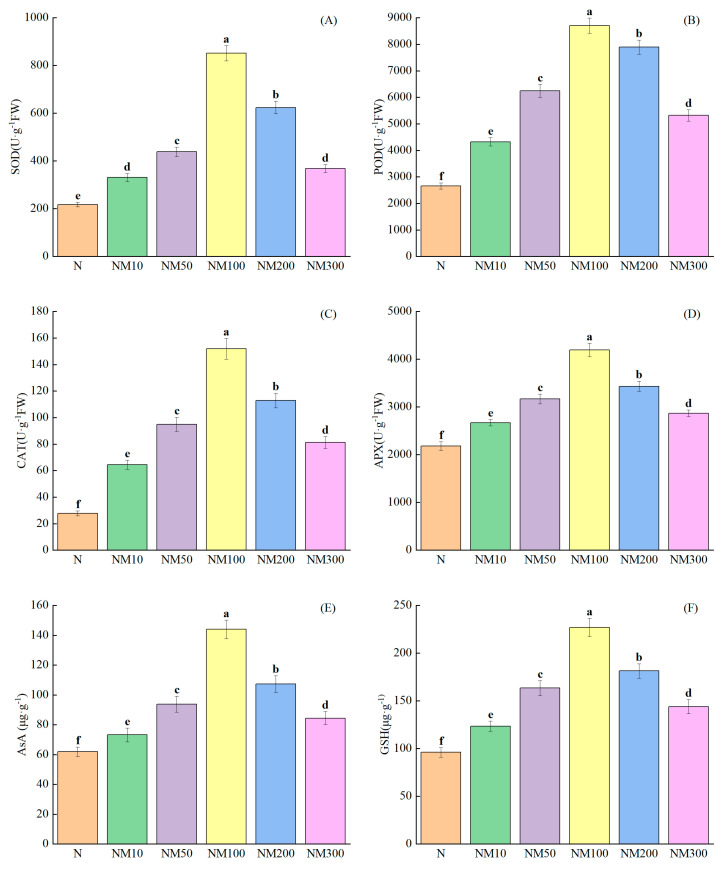
Effects of Exogenous melatonin (MT) on Antioxidant Enzyme Activities of Maize Seedlings under NaCl stress. The error lines in the figure represent the standard deviation (SD) based on three replicates (*n* = 3). Different letters indicate the significant difference among different treatments at the significant level of *p* < 0.05, as determined by one-way ANOVA. (**A**) SOD: superoxide dismutase; (**B**) POD: peroxidase; (**C**) CAT: catalase; (**D**) APX: ascorbate peroxidase; (**E**) AsA: ascorbic acid; (**F**) GSH: glutathione; N: NaCl; NM10: 150 mmol NaCl + 10 µM MT; NM50: 150 mmol NaCl + 50 µM MT; NM100: 150 mmol NaCl + 100 µM MT; NM200: 150 mmol NaCl + 200 µM MT; NM300: 150 mmol NaCl + 300 µM MT.

Salt stress significantly inhibited the level of antioxidant enzyme activities such as SOD, POD, CAT, APX, AsA, and GSH (*p* < 0.05) (Figure 5). SOD (69.97%), POD (67.6%), CAT (78.37%), APX (41.08%), AsA (44.82%), and GSH (52.36%) levels were reduced. Exogenous 100 µM MT effectively mitigated the harmful effects of salt stress, leading to significant increases of 74.47%, 71.44%, and 81.76% and 48%, 56.96%, and 57.62% in antioxidant enzyme activities in maize seedlings, respectively (*p* < 0.05). However, there was a significant decrease of 56.77%, 42.83%, 46.54%, 31.62%, 41.39%, and 36.53% in antioxidant enzyme activities compared to the NM100 (*p* < 0.05).

### 2.7. Effect of Exogenous MT on Osmoregulatory Substances in Maize Seedlings Under NaCl Stress

**Figure 6:**
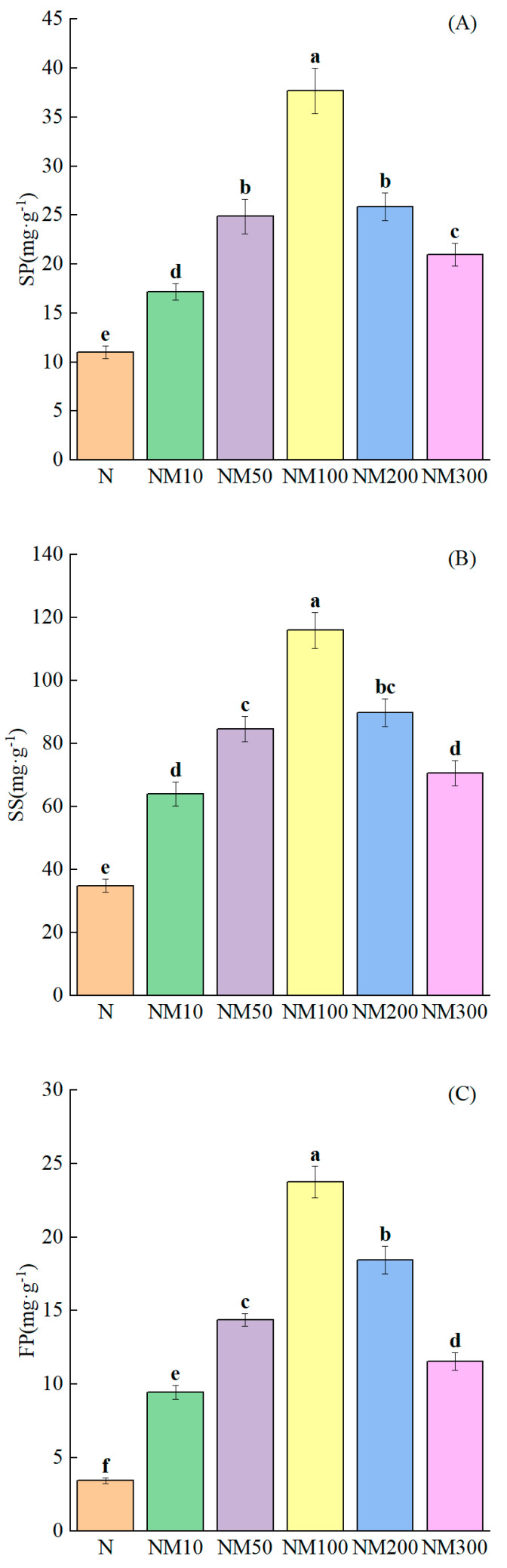
Effect of Exogenous melatonin (MT) on osmoregulatory substances in Maize Seedlings under NaCl stress. The error lines in the figure represent the standard deviation (SD) based on three replicates (*n* = 3). Different letters indicate the significant difference among different treatments at the significant level of *p* < 0.05, as determined by one-way ANOVA. (**A**) SP: soluble protein; (**B**) SS: soluble sugar; (**C**) FP: free proline. N: NaCl; NM10: 150 mmol NaCl + 10 µM MT; NM50: 150 mmol NaCl + 50 µM MT; NM100: 150 mmol NaCl + 100 µM MT; NM200: 150 mmol NaCl + 200 µM MT; NM300: 150 mmol NaCl + 300 µM MT.

NaCl stress significantly inhibited the levels of osmoregulatory substance content such as soluble protein, soluble sugar, and free proline (*p* < 0.05) (Figure 6). Soluble protein (65.08%), soluble sugar (63.41%), and free proline (83.21%) content were reduced. The application of exogenous MT ameliorated the detrimental effects of NaCl by enhancing soluble protein (35.37–70.8%), soluble sugar (45.48–69.88%), and free proline (63.94–85.61%). Notably, the most pronounced effect was observed at a concentration of 100 µM MT.

### 2.8. Effect of Exogenous MT on Ion Content in Maize Seedlings’ Roots and Leaves Under NaCl Stress

**Figure 7:**
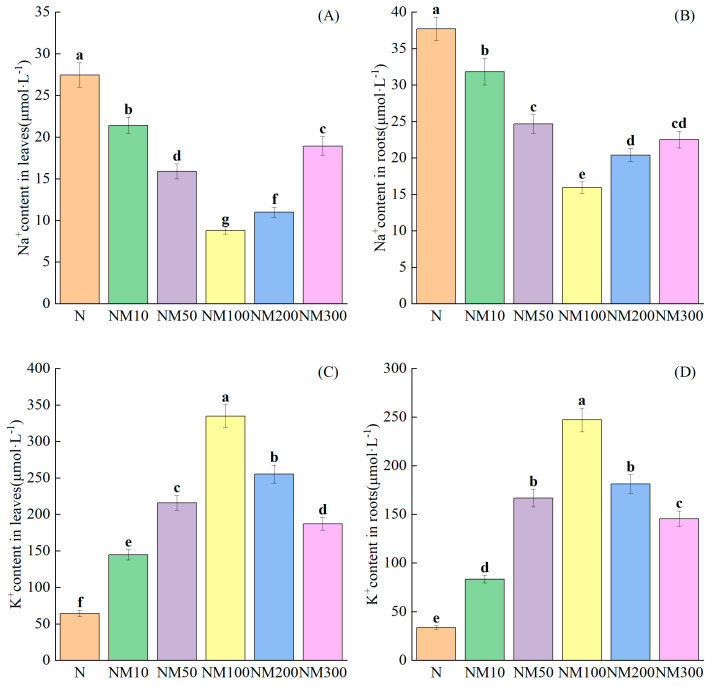
Effect of Exogenous melatonin (MT) on Ion Content in Maize Seedlings Roots and Leaves under NaCl stress. The error lines in the figure represent the standard deviation (SD) based on three replicates (*n* = 3). Different letters indicate the significant difference among different treatments at the significant level of *p* < 0.05, as determined by one-way ANOVA. (**A**) Na^+^ content in leaves; (**B**) K^+^ content in roots; (**C**) Na^+^ content in leaves; (**D**) Na^+^ content in roots. N: NaCl; NM10: 150 mmol NaCl + 10 µM MT; NM50: 150 mmol NaCl + 50 µM MT; NM100: 150 mmol NaCl + 100 µM MT; NM200: 150 mmol NaCl + 200 µM MT; NM300: 150 mmol NaCl + 300 µM MT.

NaCl stress significantly resulted in a general increase in Na^+^ and a decrease in K^+^ in roots and leaves (*p* < 0.05) (Figure 7). Exogenous MT reduced Na^+^ production in leaves and roots by 22.03–67.99% and 15.54–57.69%, respectively, and increased K^+^ production in leaves and roots by 1.24–4.19 and 1.47–6.33 times, respectively (*p* < 0.05) (Figure 7A–D).

### 2.9. Multiple Analysis of Various Indexes Changes Induced by Exogenous MT Under NaCl Stress in Maize Seeds and Seedlings

**Figure 8:**
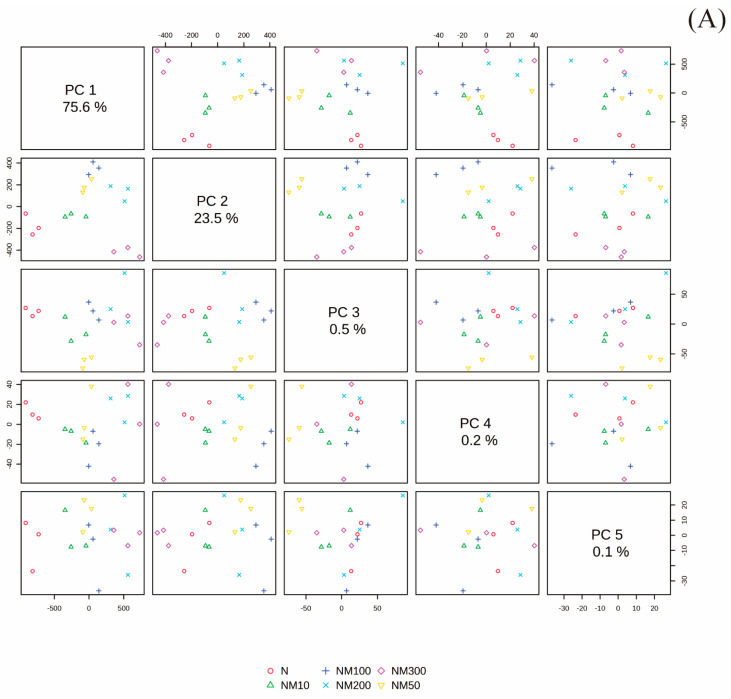

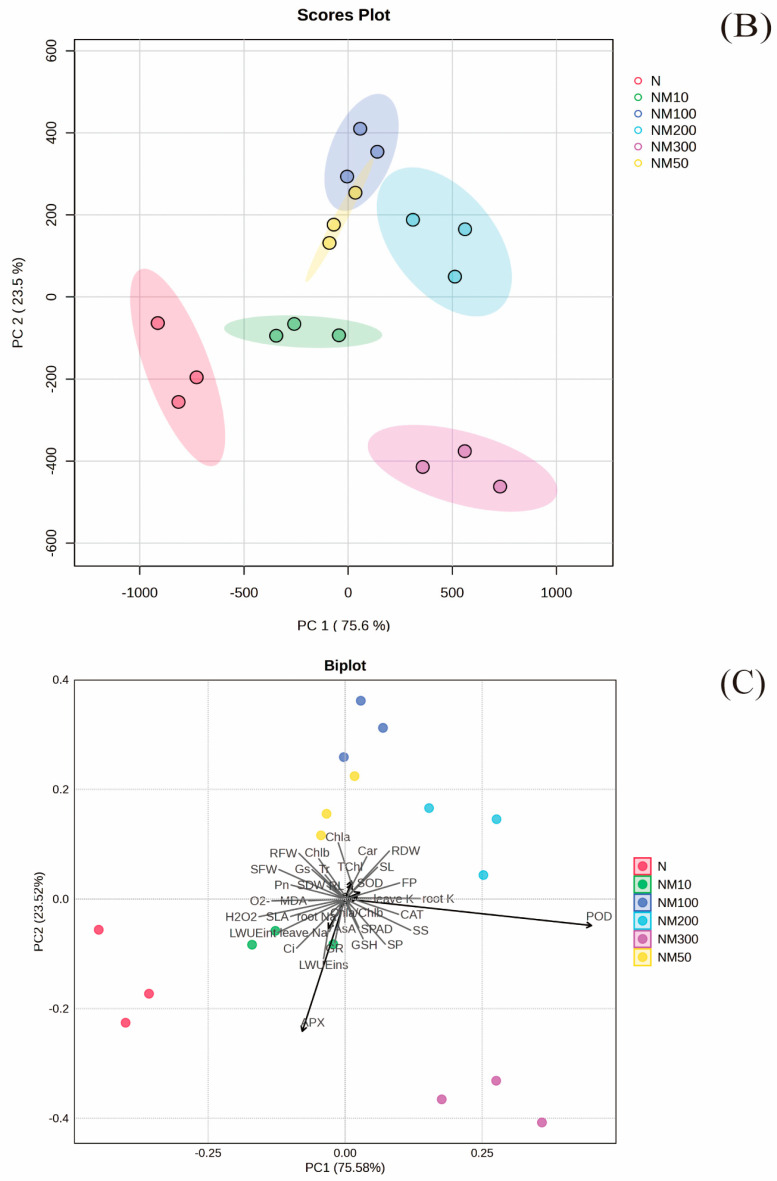
Primary component analysis (PCA) of index change of maize seeds and seedlings induced by exogenous melatonin (MT) under NaCl stress. PCA-Biplot representing (**A**) primary component, (**B**) relationship of treatments, and (**C**) relationship of indexes. GP: germination percentage; RL: root length; SL: sprout length; RFW: root fresh weight; SFW: sprout fresh weight; RDW: root dry weight; SDW: sprout dry weight; SLA: seedling leaf area; Pn: photosynthetic rate; Ci: intercellular CO_2_ concentration; Gs: stomatal conductance; Tr: transpiration rate; LWUEint: leaf intrinsic water use efficiency; LWUEins: leaf instantaneous water use efficiency. Chla: chlorophyll a; Chlb: chlorophyll b; Car: carotenoids; Tchl: Total chlorophyll content; Chla/Chlb: Ratio of chlorophyll a to chlorophyll b; SPAD: the relative chlorophyll content; MDA: malondialdehyde; H_2_O_2_:hydrogen peroxide; O_2_^−^: the rate of superoxide anion production; SOD: superoxide dismutase; POD: peroxidase; CAT: catalase; APX: ascorbate peroxidase; AsA: ascorbic acid; GSH: glutathione; SP: soluble protein; SS: soluble sugar; FP: free proline. Leave Na:Na^+^ content in leaves; root Na:Na^+^ content in roots; leave K:K^+^ content in leaves; root K: K^+^ content in roots. N: NaCl; NM10: 150 mmol NaCl + 10 µM MT; NM50: 150 mmol NaCl + 50 µM MT; NM100: 150 mmol NaCl + 100 µM MT; NM200: 150 mmol NaCl + 200 µM MT; NM300: 150 mmol NaCl + 300 µM MT.

The principal components analysis explained 99.1% of the total variance in the first two axes (PC1 and PC2) (Figure 8A). Each treatment group exhibited significant differentiation along the axes of PC1 and PC2 (Figure 8B). Malondialdehyde (MDA), hydrogen peroxide (H_2_O_2_), seedling leaf area (SLA), intercellular CO_2_ concentration (Ci), Na^+^ content in roots (root Na), Na^+^ content in leaves (leave Na), leaf instantaneous water use efficiency (LWUEins), germination percentage (GP), and ascorbate peroxidase (APX) had a high relationship with 100 mM NaCl treatment (N) and 100 mM NaCl and 10 µM MT treatment (NM10). Sprout length (SL), leaf intrinsic water use efficiency (LWUEint), ascorbic acid (AsA), glutathione (GSH), ratio of chlorophyll a to chlorophyll b (Chla/Chlb), relative chlorophyll content (SPAD), soluble sugar (SS), soluble protein (SP), K^+^ content in leaves (leave K), K^+^ content in roots (root K), and peroxidase (POD) had a high relationship with 100 mM NaCl and 300 µM MT treatment (NM300). Carotenoids (Car), root length (RL), root fresh weight (RFW), chlorophyll a (Chla), sprout fresh weight (SFW), total chlorophyll content (TChl), superoxide dismutase (SOD), and free proline (FP) had a high relationship with 100 mM NaCl and 100 µM MT treatment (NM100) and 100 mM NaCl and 200 µM MT treatment (NM200). The rate of superoxide anion production (O_2_^−^), sprout dry weight (SDW), photosynthetic rate (Pn), root dry weight (RDW), stomatal conductance (Gs), chlorophyll b (Chlb), and transpiration rate (Tr) had a high relationship with 100 mM NaCl and 100 µM MT treatment (NM100) (Figure 8B).

**Figure 9:**
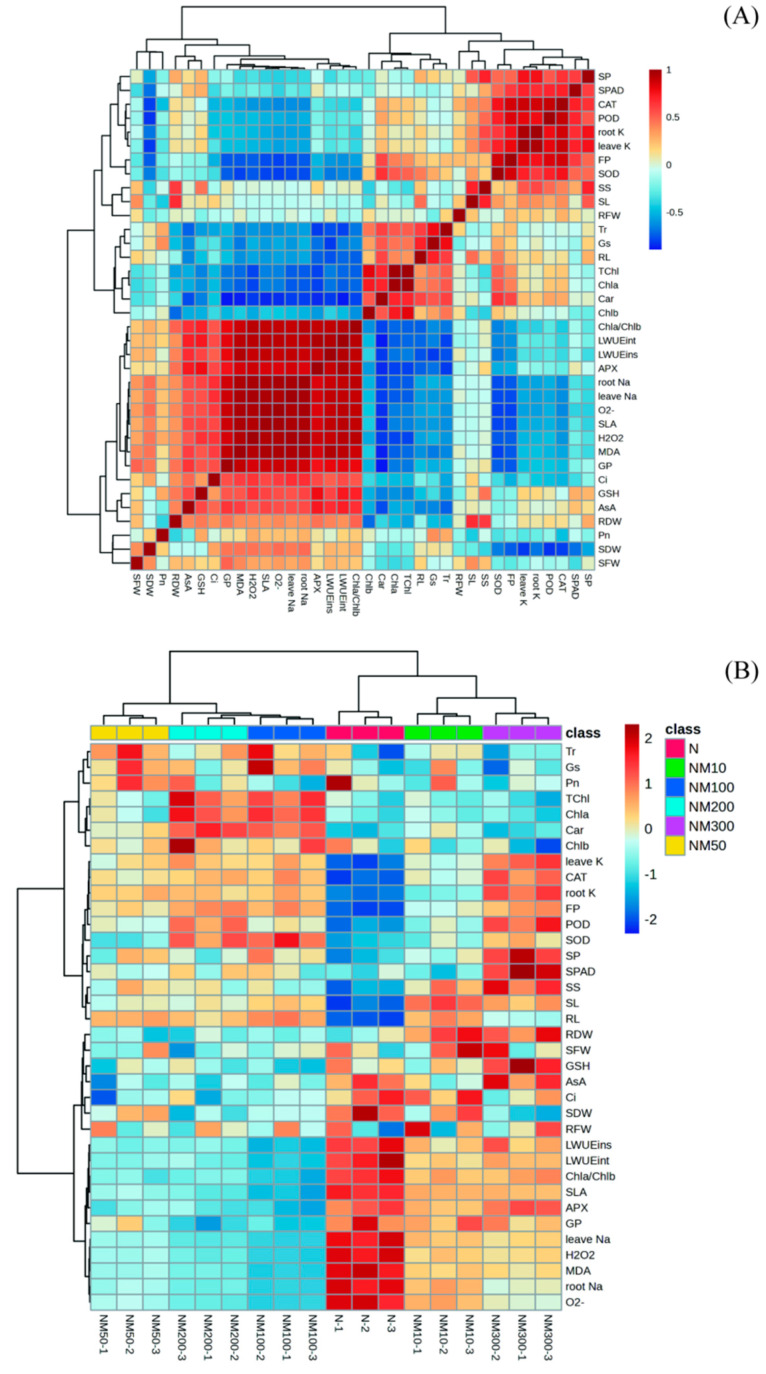
Correlation analysis (**A**) and heatmap analysis (**B**) of various index changes of maize seeds and seedlings induced by exogenous melatonin (MT) under NaCl stress. GP: germination percentage; RL: root length; SL: sprout length; RFW: root fresh weight; SFW: sprout fresh weight; RDW: root dry weight; SDW: sprout dry weight; SLA: seedling leaf area; Pn: photosynthetic rate; Ci: intercellular CO_2_ concentration; Gs: stomatal conductance; Tr: transpiration rate; LWUEint: leaf intrinsic water use efficiency; LWUEins: leaf instantaneous water use efficiency; Chla: chlorophyll a; Chlb: chlorophyll b; Car: carotenoids; Tchl: Total chlorophyll content; Chla/Chlb: Ratio of chlorophyll a to chlorophyll b; SPAD: the relative chlorophyll content; MDA: malondialdehyde; H_2_O_2_: hydrogen peroxide; O_2_^−^: the rate of superoxide anion production; SOD: superoxide dismutase; POD: peroxidase; CAT: catalase; APX: ascorbate peroxidase; AsA: ascorbic acid; GSH: glutathione; SP: soluble protein; SS: soluble sugar; FP: free proline; leave Na: Na^+^ content in leaf; root Na: Na^+^ content in root; leave K: K^+^ content in leaf; root K: K^+^ content in root; N: NaCl; NM10: 150 mmol NaCl + 10 µM MT; NM50: 150 mmol NaCl + 50 µM MT; NM100: 150 mmol NaCl + 100 µM MT; NM200: 150 mmol NaCl + 200 µM MT; NM300: 150 mmol NaCl + 300 µM MT.

Correlation (Figure 9A) and heatmap analyses (Figure 9B) elucidated the relationships among various indices. Photosynthetic Parameters (LWUEint, LWUEins), Seedling growth indications (SDW, SLA), Cell membrane damage parameters (O_2_^−^, H_2_O_2_, and MDA), Chla/Chlb, Na^+^ consent in leaves and roots, Antioxidant Enzyme Activities (GSH), and germination percentage (GP) exhibited significantly positive correlations with Pn and APX. Photosynthetic parameters (Tr, GS) and pigment (Chlb) exhibited significantly positive correlations with Pn while demonstrating a negative correlation with APX. Seedling growth indications (SL, RFW, and RL), Osmoregulatory substance (SP, FP), Antioxidant Enzyme Activities (CAT, POD, and SOD), Pigment (Chla, Car, and Tchl), and K^+^ concentration in leaves and roots exhibited significantly negative correlations with Pn and APX. Seedling growth indications (RDW, SFW), Pigment (SPAD), Osmoregulatory substance (SS), Antioxidant Enzyme Activities (ASA), and Photosynthetic Parameters (Ci) exhibited significantly positive correlations with APX while demonstrating a negative correlation with Pn.

**Figure 10:**
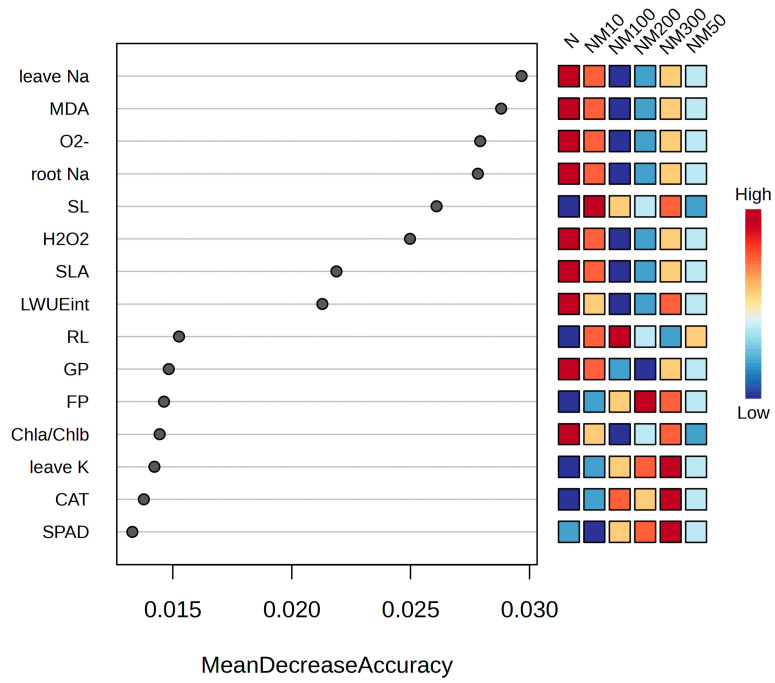
Random Forest analysis of various indexes of maize seeds and seedlings induced by exogenous melatonin (MT) under NaCl stress. SL: sprout length; SLA: seedling leaf area; RL: root length; LWUEint: leaf intrinsic water use efficiency; Chla/Chlb: Ratio of chlorophyll a to chlorophyll b; SPAD: the relative chlorophyll content; MDA: malondialdehyde; H_2_O_2_: hydrogen peroxide; O_2_^−^: the rate of superoxide anion production; CAT: catalase; FP: free proline; leave Na: Na^+^ content in leaf; root Na: Na^+^ content in root; leave K: K^+^ content in leaf; N: NaCl; NM10: 150 mmol NaCl + 10 µM MT; NM50: 150 mmol NaCl + 50 µM MT; NM100: 150 mmol NaCl + 100 µM MT; NM200: 150 mmol NaCl + 200 µM MT; NM300: 150 mmol NaCl + 300 µM MT.

The results of random forest analysis showed that, in terms of biomass accumulation, the average decline in precision of sprout length exceeded that of seedling leaf area and root length. In cell membrane damage parameters, MDA levels were higher than O_2_^−^ and H_2_O_2_ levels. In ion content, Na^+^ content in leaves was higher than Na^+^ content in roots and K^+^ content in leaves. In pigment, Chla/Chlb exceeded that of SPAD. Different treatments had significant effects on germination percentage (GP), free proline (FP), CAT, and leaf intrinsic water use efficiency (LWUEint) (Figure 10).

**Figure 11:**
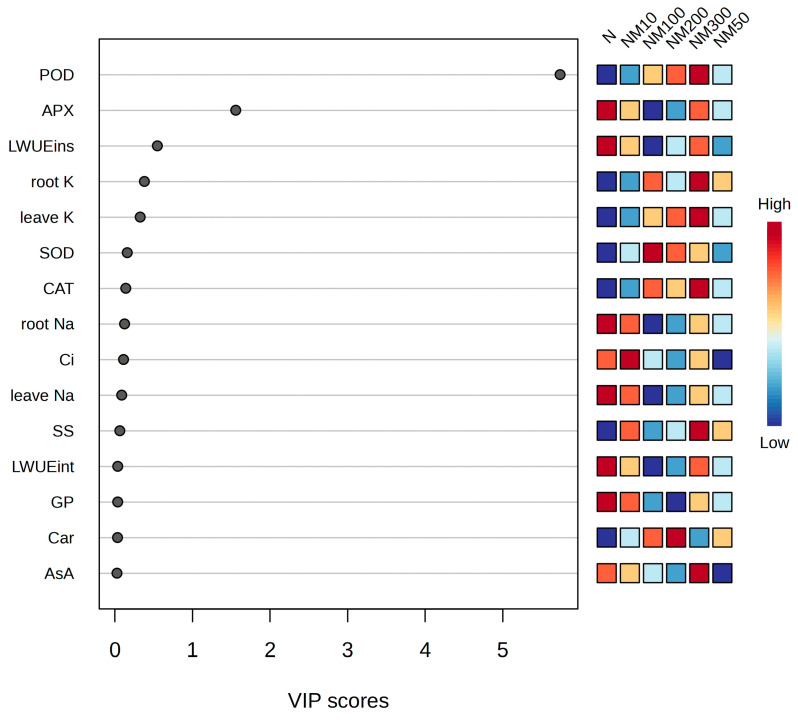
The variable importance for projection (VIP) values from the partial least squares (PLS) model and the primary factors influencing maize seeds and seedlings exposure to exogenous application of different concentrations of MT under NaCl stress conditions. (VIP > 0.1). Ci: intercellular CO_2_ concentration; LWUEint: leaf intrinsic water use efficiency; LWUEins: leaf instantaneous water use efficiency; Car: carotenoids; SOD: superoxide dismutase; POD: peroxidase; CAT: catalase; APX: ascorbate peroxidase; AsA: ascorbic acid; SS: soluble sugar; leave Na: Na^+^ content in leaf; root Na: Na^+^ content in root; leave K: K^+^ content in leaf; root K: K^+^ content in root; N: NaCl; NM10: 150 mmol NaCl + 10 µM MT; NM50: 150 mmol NaCl + 50 µM MT; NM100: 150 mmol NaCl + 100 µM MT; NM200: 150 mmol NaCl + 200 µM MT; NM300: 150 mmol NaCl + 300 µM MT.

The variable importance for projection (VIP) values derived from partial least squares (PLS) analysis revealed that some indices significantly contributed to seeds and seedlings of maize tolerance under exogenous MT application amidst salt stress, including GP, Photosynthetic parameters (Ci, LWUEins, and LWUEint), Carotenoids (Car), Antioxidant enzyme activities (SOD, POD, CAT, APX, and AsA), Soluble sugar (SS), K^+^ concentration in leaves and roots, and Na^+^ concentration in leaves and roots. Among these parameters, the POD activity is the primary factor influencing the tolerance index in seeds and seedlings (Figure 11).

## 3. Discussion

### 3.1. Effect of Exogenous Melatonin on Seed Germination and Seedling Growth of Maize Under NaCl Stress

Salt stress is an abiotic environmental stress that constrains sustainable agricultural development. Seed germination is one of the key factors affecting plant growth conditions. It has been shown that salt stress inhibits plant growth and causes yield reduction [48]. Our study showed that salt stress inhibited maize seed germination (Table 1), which confirms the adverse effects of salt stress on plant growth. Fortunately, plants have developed different strategies to cope with environmental stresses during long-term evolution [49]. It has been shown that exogenous MT improves salinity resistance in wheat and rice seedlings [50]. It has also been shown that pretreatment with 20 and 1 μM of exogenous MT increased the germination rate of seeds of cotton, wheat, and cucumber [41,50,51]. Our experimental results showed that exogenous MT could significantly alleviate the inhibitory effect of salt stress on seed germination (Figure 1). The concentration-dependent effect of MT on seed germination of maize under salt stress (Figure 1) [52] promotes growth at low concentrations and strongly inhibits growth at high concentrations. This interesting phenomenon is also reflected in the studies of Qin Bin et al. on soybean [53] and Ma Xuhui et al. on maize [54].

The root system is an important organ for plants to sense salt stress and quickly transmit this signal to the aboveground parts [55]. Our study showed that salt stress inhibited the growth of maize seedlings (Table 1) because salt stress prevents water uptake by the plant root system [56], which is consistent with the findings of Huang Chunyan et al. on sugar beet [57]. It was also found that MT could alleviate the inhibitory effect of salt stress (Table 1), indicating that MT could coordinate the growth of aboveground and belowground parts of maize, which was consistent with the results of Zuo Yuetao et al.’s study on clover [58]. In addition, it was found that MT at a concentration of 100 µM had the most significant mitigating effect on salt stress, whereas MT at 300 µM inhibited growth, suggesting that low concentration promotes growth and high concentration exacerbates the inhibition, a concentration effect similar to the results of a study on salt stress in Toon (*Toona sinensis*) seedlings [59]. Correlation analysis showed that root fresh weight was negatively correlated with the Na^+^ content of roots and leaves (Figure 9A), indicating that MT could promote growth and improve salt tolerance by reducing Na^+^ transport, a result consistent with the findings of Liu et al. [60], Zong et al. [61], and Geng et al. [62].

### 3.2. Effect of Exogenous Melatonin on Photosynthesis in Maize Seedlings Under NaCl Stress

Salt stress severely restricts photosynthesis in plants [63,64]. In our study, it found that salt stress affected photosynthetic parameters (Pn, Ci, Gs, and Tr, Figure 2A–D) in maize. Approximately 100 µM MT had the most significant effect on Gs and Tr (Figure 2C,D). Correlation analysis showed that Pn was positively correlated with Gs (Figure 9), suggesting that MT increases Gs to regulate Pn, thereby alleviating stomatal closure induced by salt stress. It has been shown in the literature that exogenous plant growth regulators can delay stomatal closure to enhance photosynthesis in edible oilseed rape, thereby reducing photosynthetic damage caused by Cd stress [61]; it has also been shown that MT can improve the Gs of maize seedlings under different soil salinities to reduce photosynthetic inhibition [65], which is consistent with our findings. MT promotes seedling growth while being able to avoid Na^+^ aggregation [66]. Salt stress causes osmotic water loss in plants, leading to physiological drought [13,67,68]. It has been shown that maize [69] and cotton [70] can increase LWUEint by reducing stomatal conductance (Gs) and photosynthetic rate (Pn). Our study found that salt stress increased LWUEint and LWUEins, while exogenous MT significantly reduced them (Figure 2E,F). Correlation analysis showed that LWUEint and LWUEins of maize seedlings were significantly positively correlated with MDA and negatively correlated with Gs (Figure 9). This suggests that exogenous MT inhibits MDA production by lowering LWUEint and LWUEins and increasing Gs, thereby improving the salt tolerance of maize seedlings. However, the intrinsic mechanism of exogenous MT regulating the function of the photosynthetic system needs to be further investigated.

Chlorophyll plays a vital role in plant tolerance to adversity [71]. It has been shown that MT inhibits the reduction of photosynthetic pigments under adversity [72,73]. In our study, we found that photosynthetic pigments were significantly reduced in maize seedlings under salt stress, whereas exogenous MT increased them (Figure 2A–F). This suggests that exogenous MT can alleviate the inhibition of photosynthesis by salt stress, which may be caused by changes in the fine structure of chloroplasts due to Na^+^ accumulation [74], which is similar to the results of Zhang et al. [75] study on the maintenance of chlorophyll stability in melon under cold stress. The results of correlation analysis showed that photosynthetic parameters (Pn, Ci, Gs, and Tr) and pigment contents (Chla, Chlb, Car, and TChl) positively affected the salt tolerance of maize seedlings, while it was negatively correlated with MDA content (Figure 9). Partial least squares analysis showed that photosynthetic parameters and pigment content played an important role in the salt tolerance of maize seedlings (Figure 11). This suggests that MT could enhance the salt tolerance of maize seedlings by increasing photosynthetic parameters and pigment content, thereby reducing oxidative damage. Thus, the enhancement of the photosynthetic system is attributed to regulating chlorophyll biosynthesis and degradation by MT, but the underlying mechanisms need to be further investigated.

### 3.3. Effect of Exogenous Melatonin on the Antioxidant System of Maize Seedlings Under NaCl Stress

Salt stress induces excessive accumulation of ROS, leading to impaired plant antioxidant systems and increased membrane peroxidation [76]; MDA reflects the degree of damage to plant cell membranes under stress conditions [77]; and H_2_O_2_ and O_2_^−^ are key signaling molecules reflecting plant cellular senescence and resistance responses to adversity stress [78]. In our study, we found that the elevation of MDA in maize seedlings was consistent with salt stress-induced H_2_O_2_ and O_2_^−^ accumulation, providing strong evidence that excessive ROS lead to salt stress-induced cell membrane damage (Figure 5A–C). MT is an antioxidant that directly scavenges the accumulation of excessive ROS through a cascade reaction [79]. Mancher [80] et al. MT protects plants from oxidative damage and also scavenges free radicals and excess ROS. Our results showed that exogenous MT significantly reduced the levels of MDA, H_2_O_2_, and O_2_^−^ and reduced the accumulation of excess ROS in maize seedlings under salt stress (Figure 3A–C). This is consistent with previous findings that MT application inhibited ROS accumulation and induced membrane peroxidation responses in tomato seedlings [81], watermelon [82], and kiwifruit [83] under different abiotic factors of stress.

The antioxidant defense system of plants includes both enzymatic and non-enzymatic systems, which mainly act to scavenge ROS produced intracellularly under adversity [78]. Our study found that MT enhanced the antioxidant enzyme activities (SOD, POD, CAT, and APX) in maize seedlings under salt stress (Figure 5A–D), and previous studies have shown that MT improved the antioxidant enzyme activities in tomato seedlings under salt stress [84]. Meanwhile, similar results were found in abiotic stress studies in tomatoes [81], cucumbers [85], and watermelon [86], which is consistent with the results of this study. Correlation analysis showed that H_2_O_2_, MDA, and O_2_^−^ levels in maize seedlings were negatively correlated with POD, SOD, and CAT (Figure 9) and positively correlated with APX (Figure 9), suggesting that low concentrations of MT reduced enzyme activities, since MT has antioxidant properties that enhance the ability to scavenge ROS and improve the plant’s resistance to oxidative stress. Partial least squares analysis also showed that POD, SOD, CAT, and APX activities affected salt tolerance in maize seedlings (Figure 10). This suggests that MT increases antioxidant enzyme activities and reduces membrane lipid peroxidation, thereby protecting maize plants from salt stress damage. Similar findings were found in Cd-stressed wheat [60] and edible oilseed rape [61], as well as in salt-stressed maize [5].

AsA and GSH are non-enzymatic antioxidants, AsA is a small-molecule antioxidant and electron donor for redox reactions that acts directly on reactive oxygen species [87], and GSH regulation of H_2_O_2_ levels affects AsA regeneration to activate plant antioxidant defense [88,89]. Our study showed that salt stress reduced AsA and GSH levels in maize seedlings. Interestingly, changes in AsA and GSH content showed a concentration effect with the MT, with the most significant effect at a concentration of 100 µM (Figure 5E,F). This suggests that low concentrations of MT can increase the AsA and GSH contents of maize seedlings to avoid salt stress-induced oxidative damage, and high concentrations of MT aggravate salt stress-induced damage. Correlation analysis showed a positive correlation between AsA, GSH, and MDA (Figure 8), which indicates that the appropriate concentration of MT can increase the contents of AsA and GSH to alleviate oxidative damage and maintain the redox balance of maize seedlings. In conclusion, the contents of AsA and GSH under adversity stress depended on the concentration of MT applied.

Carotenoids, potent antioxidants, can promote free radical detoxification by quenching singlet oxygen [90]. Exogenously applied β-carotene alleviated the toxic effects of bisphenol A (BPA) exposure on tobacco plants, as evidenced by increased biomass, chlorophyll content, and decreased MDA levels [91]. In our study, we found that salt stress reduced the carotenoid content of maize seedlings, but the concentration effect of carotenoid content with changes in MT (Figure 3C). In addition, we found that the carotenoid content was negatively correlated with the MDA level (Figure 9), which was attributed to the fact that exogenous MT could increase the carotenoid content of maize seedlings to scavenge the over accumulated ROS, thus alleviating the oxidative damage induced by salt stress. This suggests that MT can regulate antioxidant levels to reduce salt stress-induced antioxidant damage.

Osmoregulation plays an important role in maintaining intracellular stability and protecting cells from abiotic stress damage [92,93]. Salt stress causes osmotic stress injury in plants, inducing changes in intra- and extracellular osmotic pressure, leading to ion toxicity and osmotic imbalance. Plants mitigate the damage by spontaneously increasing osmoregulatory substances to balance water potential [94]. In this study, we found that salt stress decreased SP, SS, and FP in maize seedlings, which was attributed to the destruction of the cellular structure by salt stress, thus leading to a decrease in the content of osmoregulatory substances, which was in agreement with the findings of Guo Yuanhang [95] et al. on salt stress in soybean. Exogenous MT has been shown to regulate osmotic substances (SP, SS, and FP) to alleviate osmotic stress mainly at the cellular level [96]. In our study, we found that exogenous MT increased SP, SS, and FP in maize seedlings (Figure 6A–C), which was most pronounced at a concentration of 100 µM, because MT enables plants to actively accumulate osmoregulatory substances to increase cytosol concentration, reduce osmotic potential, and alleviate the cellular osmoregulatory imbalance caused by stress [97]. It is similar to the findings of Li He [98] et al., Qin Bin [53] et al., and Huang Rong [99] et al. in soybean salt stress. However, the content of osmoregulatory substances gradually decreased with increasing MT concentration (Figure 6A–C), while the levels of membrane plasma peroxides (MDA, H_2_O_2,_ and O_2_^−^) increased (Figure 4). Correlation analysis showed that the content of osmoregulatory substances (SP, SS, and FP) was negatively correlated with the levels of membrane plasma peroxides (MDA, H_2_O_2,_ and O_2_^−^) (Figure 9). Least partial squares analysis (PLS) showed that SS content affected the salt tolerance of maize seedlings (Figure 11). This suggests that appropriate concentrations of MT can improve osmoregulation in maize seedlings, and this concentration effect was similarly found in salt stress studies of watermelon [82] and parsnip [59] seedlings.

### 3.4. Effect of Exogenous Melatonin on the Ion Content of Roots and Leaves of Maize Seedlings Under NaCl Stress

In our study, we found that Na^+^ content increased and K^+^ content decreased in leaves and roots of maize seedlings under salt stress (Figure 7A–D), which is similar to the findings of Li, X. et al. [100] in rice and Ren, J. et al. [101] in maize. It is because Na^+^ is sensed by glucuronosyltransferase, activates Ca^2+^ channels, and increases Ca^2+^ influx into the cytosol [102]. We found that MT reduced Na^+^ content and increased K^+^ content in leaves and roots of maize seedlings under salt stress, while [K^+^]/[Na^+^] was elevated (Figure 7A–D). We also found that the Na^+^ content in roots of maize seedlings was significantly higher than that in leaves when treated with 100 µM MT (Figure 6A,B). This is due to the fact that MT promotes the transport of Na in the root system and maintains the ionic balance to avoid the inhibitory effect of salt stress. It can be suggested that MT may first have an ameliorating effect on roots, and the Na^+^ content in leaves only gradually increased as the nutrient solution flowed and circulated in stems and leaves. Kaya et al. [103] found in wheat that MT treatment increased Ca^2+^ and K^+^, decreased MDA and H_2_O_2_, and promoted plant growth in the leaves of Cd-stressed wheat plants, which was similar to our findings. Correlation analysis showed that Na^+^ content in roots and leaves was positively correlated with membrane lipid peroxides (MDA, H_2_O_2_, and O_2_^−^) and negatively correlated with osmoregulatory substances (SP, SS, and FP) (Figure 8), and K^+^ content in roots and leaves was negatively correlated with membrane lipid peroxides (MDA, H_2_O_2_, and O_2_^−^) and positively correlated with osmoregulatory substances (SP, SS, and FP) (Figure 8). Least partial squares analysis showed that K^+^ content and Na^+^ content in roots would have an effect on salt tolerance of maize seedlings (Figure 10). This suggests that the appropriate concentration of MT can regulate the ion content in the roots and leaves of maize seedlings to alleviate salt stress injury.

**Figure 12:**
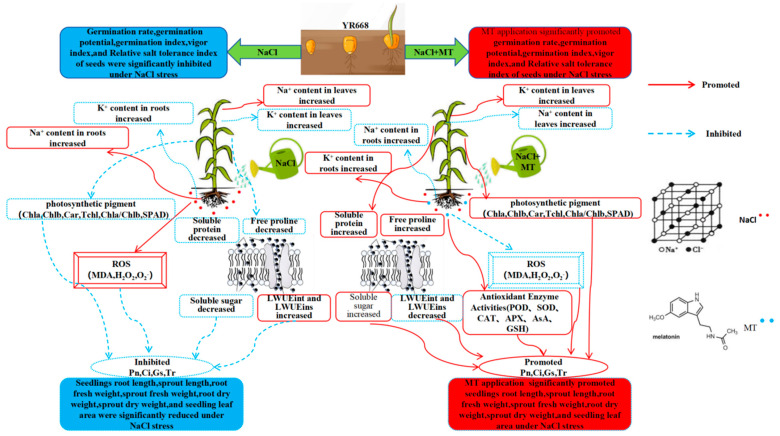
Schematic model of response mechanism for exogenous melatonin (MT) alleviates NaCl injury in maize seedlings. Red solid arrows indicated promoting effects; Blue dashed arrows indicated inhibited effects. Pn: photosynthetic rate; Ci: intercellular CO_2_ concentration; Gs: stomatal conductance; Tr: transpiration rate; LWUEint: leaf intrinsic water use efficiency; LWUEins: leaf instantaneous water use efficiency; Chla: chlorophyll a; Chlb: chlorophyll b; Car: carotenoids; Tchl: Total chlorophyll content; Chla/Chlb: Ratio of chlorophyll a to chlorophyll b; SPAD: the relative chlorophyll content; MDA: malondialdehyde; H_2_O_2_: hydrogen peroxide; O_2−_: the rate of superoxide anion production; SOD: superoxide dismutase; POD: peroxidase; CAT: catalase; APX: ascorbate peroxidase; AsA: ascorbic acid; GSH: glutathione; SP: soluble protein; SS: soluble sugar; FP: free proline.

Taken together, our data provide detailed protective evidence for exogenous MT in maize under salt stress by investigating seed germination index, seedling growth index, photosynthesis, antioxidant system, and root and leaf ion content. This protective role may be involved in the activation of the antioxidant defense system, the elimination of ROS, and the protection of photosynthetic apparatus. For this purpose, we try to construct a possible mechanistic map of the role of MT in the improvement of salt resistance in maize seedlings under NaCl stress (Figure 12), which will provide a basis for a systematic understanding of molecular mechanisms on exogenous MT alleviating salt injury in maize.

## 4. Materials and Methods

### 4.1. Seed Germination Test

Full maize seeds (YR668 variety) were handpicked and sterilized by soaking in 10% NaCl for 10 min, then washed five times with distilled water and soaked for 3 h. Seeds were scattered in Petri dishes (15 cm in diameter, using two layers of filter paper as germination beds) with 20 seeds per dish, maintaining a spacing of 1.5 cm. A salt concentration of 150 mM (NaCl), as determined in preliminary experiments, was used as a threshold value. Five MT treatments were set up: 0, 10, 50, 100, 200, and 300 µM. The treatment solutions consisted of NaCl and MT-labeled N, NM10, NM50, NM100, NM200, and NM300. Three replicates were made for each treatment. Hoagland nutrient solution was used as a control (labeled Control). Ten milliliters of treatment solution was added to each petri dish and placed in an incubator at 25 °C. The filter paper and the treatment solution were replaced daily. The filter paper and treatment solution were changed daily to maintain a consistent osmotic potential and to promote germination.

### 4.2. Seedling Hydroponic Experiment

Seven days post-germination, transfer uniformly germinated seeds to culture pots for further growth, planting 32 seedlings per pot. Each pot receives 3 L of treatment solution, changed every three days. Concentrations of NaCl and MT in the mixed treatment solution remain identical to those in the seed germination test.

### 4.3. Determination of Germination Indicators

Record the number of germinated seeds at a fixed time each day during cultivation (germination criterion: radicle length greater than 2 mm). Calculate germination percentage on day 7.

Germination percentage (G%) = (total number of seeds germinated within 7 days/total number of tested seeds) × 100%. [104].

### 4.4. Determination of Physiological Indicators for Seedling Growth

Fifteen days post salt stress, randomly selected five seedlings with consistent growth from each treatment. We measured the root length (RL) and sprout length (SL). We rinsed the seedlings five times with distilled water, dried them with filter paper, and measured root fresh weight (RFW) and sprout fresh weight (SFW). We then placed samples in an oven, blanched them at 105 °C for 30 min, and dried them at 80 °C until constant weight was achieved. We recorded these as root dry weight (RDW) and sprout dry weight (SDW), repeating each treatment three times [105]. The seedling leaf area (SLA) was calculated as follows:

SLA = leaf length × leaf width × 0.75(1)

### 4.5. Determination of Photosynthetic Parameters and Chlorophyll

Photosynthetic parameters of maize seedlings, including photosynthetic rate (Pn), intercellular CO_2_ concentration (Ci), stomatal conductance (Gs), and transpiration rate (Tr), were measured using a portable photosynthesis system (Li-6400, LICOR Inc., Lincoln, NE, USA). Leaf intrinsic water use efficiency (LWUEint) and leaf instantaneous water use efficiency (LWUEins) were calculated using the method described by Liu et al. [106].

The relative chlorophyll content (SPAD) value of the third leaf of maize seedlings under all treatments was determined using the chlorophyll meter (SPAD-502; KonicaMinolta Sensing, Inc., Tokyo, Japan).

The 1.0 g fresh leaves sample from each treatment was placed in 10 mL of 95% alcohol (*v*/*v*) for 48 h in darkness. The absorbance values of the extracting solution were then measured using a multifunctional enzyme marker model (SynergyHTX; BioTek Instruments, Inc., Winooski, VT, USA) at 665 nm, 649 nm, and 470 nm. The concentrations of chlorophyll a (Chla), chlorophyll b (Chlb), carotenoids (Car), Chla/Chlb, and total chlorophyll (TChl).

The sum of chlorophyll a and chlorophyll b was calculated as follows:

Ca = 13.95 × A665 − 6.88 × A649(2)

Cb = 24.96 × A649 − 7.32 × A665(3)

Cc = 1000 × A470 − 2.05 × Ca − 114 × Cb(4)

The contents of these pigments were then calculated as follows:

Chlorophyll a content (μg/g) = Ca × Vt × n/FW × 1000(5)

Chlorophyll b content (μg/g) = Cb × Vt × n/FW × 1000(6)

Carotenoid content (μg/g) = Cc × Vt × n/FW × 1000(7)

Total chlorophyll content (μg/g) = Chlorophyll a content + Chlorophyll b content(8)

Chla/Chlb = Chlorophyll a content/Chlorophyll b content(9)

where Ca, Cb, and Cc represented the concentrations of chlorophyll a (Chl a), chlorophyll b (Chl b), and carotenoids (Car), respectively. FW referred to the fresh weight of the sample (g), Vt was the total volume of extracting solution (mL), and n was the dilution factor of extracting solution.

### 4.6. Determination of Membrane Damage Index

Determination of malondialdehyde (MDA) was conducted using the thiobarbituric acid method [107]. The hydrogen peroxide (H_2_O_2_) content was determined according to Jessup, Dean & Gebicki [108]. The rate of superoxide anion production (O_2_^−^ production rate) was determined according to the method described by Lin et al. [109].

### 4.7. Determination of Physiological and Biochemical Indicators

Superoxide dismutase (SOD) activity was measured using the Nitro-Blue Tetrazolium (NBT) method; peroxidase (POD) activity was determined by the guaiacol method; and catalase (CAT) activity was assessed using UV absorption [110].

Ascorbate peroxidase (APX) activity was determined according to the method described by Nakano & Asada [111]. The reduced ascorbic acid (AsA) content was measured according to the method of Chen et al. [112]. The glutathione (GSH) contents were quantified according to the procedure specified by Guri [113].

Soluble protein (SP) content was measured using the Coomassie Brilliant Blue G250 staining method; soluble sugar (SS) was quantified by the anthrone colorimetric method; and free proline (FP) levels were evaluated using the ninhydrin colorimetric method [90].

### 4.8. Determination of Ion Content

Dried samples were digested using the H_2_SO_4−_H_2_O_2_ method in a Topwave microwave digestion analyzer (Analytik, Jena, Germany). The digestion process was conducted in accordance with the operational procedures of the microwave digestion instrument. K and Na standard solutions were purchased from the National Center for Analysis and Testing of Nonferrous Metals and Electronic Materials. The contents of K and Na were determined by a flame spectrophotometer (TAS-990, Beijing Puyang General Instrument Co., Ltd., Beijing, China) [114].

### 4.9. Statistical Analyses

Data were processed using Microsoft Excel 2021, one-way analysis of variance (ANOVA) was performed using SPSS 25.0 software, multiple comparisons were performed using Duncan’s Test for Multiple Comparisons (Duncan’s Test), principal component and correlation analysis plots were plotted using Origin 2021 analysis, and randomized Forest (RF) and Partial Least Squares (PLS) analysis were performed using Metaboanalyst.

## 5. Conclusions

In this study, the application of exogenous MT demonstrated its efficacy in mitigating the adverse effects of salt stress on maize seeds and seedlings. Salt stress inhibited various physiological and biochemical indices such as seed germination parameters, seedling growth indices, photosynthetic properties, and antioxidant defense mechanisms. However, MT application enhanced the photosynthetic system, reduced sodium (Na) uptake, lowered malondialdehyde (MDA), H_2_O_2_, and O_2_^−^ levels, and enhanced antioxidant effects, resulting in increased salt tolerance and improved growth of maize seedlings under salt stress. Among the various concentrations of MT treatments, 100 µM MT showed the best potential to mitigate the deleterious effects of salt stress on maize seeds and seedlings. Therefore, future research should focus on the long-term impacts of MT application under field conditions to improve the sustainability and practical application of MT in various agricultural environments.

## Figures and Tables

**Table 1:** Effect of Exogenous melatonin (MT) on the Seedling growth indicators of Maize under NaCl stress.

Treatment	The Seedling Growth Indicators						
	RL (cm)	SL (cm)	RFW (g)	SFW (g)	RDW (g)	SDW (g)	SLA (cm^2^)
N	0.70 ± 0.02 e	0.31 ± 0.03 e	0.24 ± 0.03 d	0.37 ± 0.03 d	0.024 ± 0.002 d	0.043 ± 0.003 d	18.53 ± 0.22 d
NM10	1.81 ± 0.06 c	0.98 ± 0.03 c	0.34 ± 0.05 c	0.52 ± 0.04 c	0.041 ± 0.003 bc	0.053 ± 0.006 c	20.95 ± 0.34 c
NM50	2.48 ± 0.06 b	1.04 ± 0.03 c	0.46 ± 0.05 b	0.68 ± 0.04 b	0.043 ± 0.003 bc	0.071 ± 0.003 b	23.73 ± 0.48 b
NM100	3.78 ± 0.04 a	1.68 ± 0.04 a	0.68 ± 0.04 a	0.98 ± 0.05 a	0.064 ± 0.004 a	0.096 ± 0.003 a	27.91 ± 0.43 a
NM200	2.60 ± 0.09 b	1.16 ± 0.06 b	0.50 ± 0.04 b	0.71 ± 0.05 b	0.047 ± 0.005 b	0.066 ± 0.006 b	24.28 ± 0.39 b
NM300	1.45 ± 0.04 d	0.86 ± 0.04 d	0.33 ± 0.03 c	0.49 ± 0.04 c	0.040 ± 0.003 c	0.042 ± 0.003 d	20.25 ± 0.27 c

Data are shown as means ± SD. Different letters indicate the significant difference among different treatments at the significant level of *p* < 0.05, as determined by one-way ANOVA. RL: root length; SL: sprout length; RFW: root fresh weight; SFW: sprout fresh weight; RDW: fresh weight of root; SDW: sprout dry weight (SDW); SLA: seedling leaf area. N: NaCl; NM10: 150 mmol NaCl + 10 µM MT; NM50: 150 mmol NaCl + 50 µM MT; NM100: 150 mmol NaCl + 100 µM MT; NM200: 150 mmol NaCl + 200 µM MT; NM300: 150 mmol NaCl + 300 µM MT.

## Data Availability

Data are contained within the article.
